# The Syvn1 inhibits neuronal cell ferroptosis by activating Stat3/Gpx4 axis in rat with spinal cord injury

**DOI:** 10.1111/cpr.13658

**Published:** 2024-05-27

**Authors:** Shining Xiao, Yu Zhang, Shijiang Wang, Jiaming Liu, Fan Dan, Feng Yang, Shue Hong, Ning Liu, Yujia Zeng, Ke Huang, Xinsheng Xie, Yanxin Zhong, Zhili Liu

**Affiliations:** ^1^ Department of Orthopedics the First Affiliated Hospital, Jiangxi Medical College, Nanchang University Nanchang People's Republic of China; ^2^ Jiangxi Provincial Key Laboratory of Spine and Spinal Cord Diseases Nanchang People's Republic of China; ^3^ Medical Innovation Center the First Affiliated Hospital, Jiangxi Medical College, Nanchang University Nanchang People's Republic of China; ^4^ Department of Spine Surgery Ganzhou People's Hospital Ganzhou People's Republic of China; ^5^ Department of Rehabilitation Medicine the First Affiliated Hospital, Jiangxi Medical College, Nanchang University Nanchang People's Republic of China

## Abstract

Spinal cord injury (SCI) leads to secondary neuronal death, which severely impedes recovery of motor function. Therefore, prevention of neuronal cell death after SCI is an important strategy. Ferroptosis, a new form of cell death discovered in recent years, has been shown to be involved in the regulation of SCI. However, the role and potential mechanisms of ferroptosis in secondary SCI are not fully understood. In this study, we report that the E3 ubiquitin ligase Syvn1 suppresses ferroptosis and promotes functional recovery from SCI in vitro and in vivo. Mechanistically, screened with bioinformatics, immunoprecipitation, and mass spectrometry, we identified Stat3, a transcription factor that induces the expression of the ferroptosis inhibitor Gpx4, as a substrate of Syvn1. Furthermore, we identified neurons as the primary cellular source of Syvn1 signalling. Moreover, we determined the binding domains of Syvn1 and Stat3 in HEK 293 T cells using full‐length proteins and a series of truncated Flag‐tagged and Myc‐tagged fragments. Furthermore, we created the cell and animal models with silencing or overexpression of Syvn1 and Stat3 and found that Syvn1 inhibits neuronal ferroptosis by stabilizing Stat3, which subsequently activates the ferroptosis regulator Gpx4 in SCI. In summary, the Syvn1‐mediated Stat3/Gpx4 signalling axis attenuates neuronal ferroptosis, reduces neuronal death, and promotes SCI repair. Therefore, our findings provide potential new targets and intervention strategies for the treatment of SCI.

## Graphical abstract

Syvn1 interacts with Stat3 to regulate its ubiquitination and stability, which in turn activates Gpx4 expression and ameliorates ferroptosis in neurons after SCI.

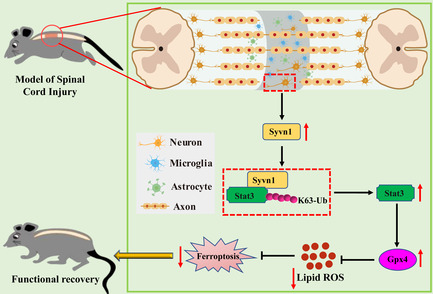

## 1 INTRODUCTION

Spinal cord injury (SCI) is a severe neurological condition associated^1^, ^2^ that triggers secondary events, such as oxidative stress and axonal damage, which ultimately leads to tissue damage neuronal death, and long‐term or permanent loss of sensory and motor function.^3^ There is no effective treatment for SCI, and current therapeutic strategies focus on preventing or reducing this secondary damage.^4^ This is because the mechanisms underlying the secondary injury to neurons after SCI still need to be fully understood. The associated processes must be clarified to achieve the goal of SCI repair.

Ferroptosis is a form of cell death induced by dysregulated iron metabolism that leads to the accumulation of lipid peroxides and, consequently, cell membrane damage.^5^ Studies have shown that ferroptosis plays an important role in the pathogenesis of SCI.^6^ After SCI, the concentration of iron ions in the injured spinal cord area markedly increases.^7^ This results in the generation of a large amount of ROS and oxidative stress‐related products through the Fenton reaction, which in turn leads to increased neuronal death and damage.^8^, ^9^ Additionally, inhibition of ferroptosis was observed to be effective in increasing neuronal survival and promoting functional recovery in SCI models.^10^ However, the mechanisms that underlie the regulation of ferroptosis in neurons after SCI are still unclear.

Protein stabilization mediated by the ubiquitin‐proteasome pathway is an important post‐translational modification process and a major molecular mechanism affecting a variety of neurodegenerative diseases.^11^, ^12^ Ubiquitin regulation of protein stabilization is a cascade reaction involving multiple enzymes.^13^ Among them, ubiquitin ligase E3 can play an important role in the ubiquitination process by binding to the direct substrate, while deubiquitinases (DUBs) can dissociate ubiquitin from the ubiquitin‐labelled substrate proteins and reverse the ubiquitination process.^14^ Thus, ubiquitin ligase E3 and DUBs act as key regulatory proteins in the ubiquitination process. Syvn1 is an E3 ubiquitin ligase that is highly expressed in brain neurons.^15^ The knockdown of Syvn1 can lead to the accumulation of amyloid beta and the exacerbation of neuronal apoptosis in an Alzheimer's model,^16^ suggesting the importance of Syvn1‐mediated protein stabilization in the nervous system. Although Syvn1 has been implicated in neurological diseases, the specific underlying molecular mechanisms have yet to be elucidated, especially in traumatic SCI.

In this study, we found that the E3 ligase Syvn1, a ferroptosis‐associated protein, is involved in the regulation of neuronal ferroptosis and improves functional recovery after SCI. To further elucidate the specific molecular mechanism process by which Syvn1 improves neuronal ferroptosis after SCI, we found that the E3 ligase Syvn1 can interact with the substrate Stat3 to enhance K63‐linked polyubiquitin chain modification and maintain its stability. Additionally, the accumulation of Stat3 can induce activation of Gpx4 at the transcriptional level. Our findings elucidate that Syvn1 regulates neuronal ferroptosis through the Stat3/Gpx4 axis and may provide a new therapeutic option for SCI intervention.

## 2 MATERIALS AND METHODS

### 2.1 SCI model

Female Sprague‐Dawley (SD) rats (Vital River, China) weighing 200–220 g were anaesthetised with 1% sodium pentobarbital (40 mg/kg) as previously described. Subsequently, a laminectomy was performed at the T9 vertebral body to expose the spinal cord, which was subsequently clamped with vascular clips for 10 s to create a rat SCI model. The bladder was emptied manually three times a day until the bladder function of the rats recovered.

### 2.2 Assessment of functional behaviour

Functional recovery of the hindlimbs of rats was assessed using the Basso Beattie Bresnahan (BBB) locomotor recovery scale within 28 days of surgery. For footprint analysis, red and blue ink were painted on the front and hind paws of the rats, respectively, and the animals were then allowed to walk on white paper for footprint analysis. Hind limb coordination was evaluated by observing the footprints. Additionally, motor evoked potentials in the tibialis anterior muscle of the rats were measured 28 days after SCI using electromyography.

### 2.3 Cell culture

VSC 4.1 neurons were purchased from Otwo Biotech (Shenzhen, China). BV2 microglia, HEK 293T cells, and bEnd.3 brain‐derived endothelial cells were obtained from Procell (Wuhan, China). Upon receipt, the cells were cultured in high‐glucose DMEM (Gibco, USA) supplemented with 10% FBS (Gibco, USA).

For the isolation and culture of primary cortical neurons, we selected SD rats within 24 h of birth. Briefly, we will dissociate the cerebral cortex from the brain tissue, cut it into pieces, add 0.25% trypsin for digestion, and then use DMEM/F12 medium (Gibco, USA) containing 10% FBS to terminate the digestion. Then filter with a 40 μm mesh, centrifuge, resuspend the cells in Neurobasal Medium (Gibco, USA) containing 2% B27 (Gibco, USA), 1% Glutamax (Gibco, USA) and Glutamate (25 μM) (Sigma, USA), and seed them in a culture plate pre‐coated with polylysine (Beyotime, China). The medium was then changed every 2 days with Neurobasal Medium containing 2% B27 and 1% Glutamax.

### 2.4 Cell viability assay

We seeded neurons in 96‐well plates, incubated them with 10% Cell Counting Kit‐8 (CCK‐8) solution (Beyotime, China) for 2 h, and then measured their absorbance at 450 nm using a microplate reader (Thermo Scientific, USA) to assess cell viability.

### 2.5 Cell death detection

We stained neurons with PI (Solarbio, China) and/or 6‐diamidino‐2‐phenylindole (DAPI) (Beyotime, China) for 30 min. Cell death was observed under an inverted fluorescence microscope and flow cytometry (Agilent, USA).

### 2.6 Ferroptosis level

Malonaldehyde (MDA), GSH, and Fe^2+^ levels in cell or tissue lysates were measured using MDA (Beyotime, China), GSH (Sigma, USA), and Fe^2+^ Concentration assay kits (Dojindo, Japan) according to the instructions of the respective manufacturers.

### 2.7 Dihydroethidium (DHE) assay

Neurons or spinal cord tissues were incubated with DHE fluorescent probe (Beyotime) at 37°C for 30 min in the dark, following which ROS levels were observed by flow cytometry or inverted fluorescence microscopy.

### 2.8 Transmission electron microscopy

We digested the neurons with trypsin, then fixed the cells with 2.5% glutaraldehyde and 1% osmium acid, respectively, followed by dehydration by replacing the 50%, 70%, 80%, 90%, 100%, and 100% ethanol solutions sequentially. The samples were then embedded and ultrathin sections were made for observation and imaging using a transmission electron microscope (FEI Tecnai, USA).

### 2.9 Co‐immunoprecipitation (Co‐IP) detection

Proteins were extracted from the cells using immunoprecipitation lysis buffer (Thermo Scientific, USA), followed by overnight incubation with 1 μg of the corresponding antibody. Next day, proteins were incubated with Protein A/G Magnetic Beads (MCE, USA) for 2 h. After washing with immunoprecipitation buffer, SDS–PAGE loading buffer (Beyotime, China) was added for immunoblotting.

### 2.10 IP/MS assay

As described above, we first obtained total proteins from neurons. Then we incubated 1 μg antibody (anti‐Syvn1 or anti‐IgG) and Protein A/G Magnetic Beads for IP experiments. Finally, MS was performed to identify the proteins that bind to Syvn1.

### 2.11 Molecular docking

Syvn1 (UniProt ID: F7FG68) and Stat3 (UniProt ID: P52631) were selected from rat species, their amino acid sequences were obtained from the UniProt website, and their three‐dimensional structures were predicted by AlphaFold. Additionally, PyMOL (version 2.5) and PDBePISA were applied to evaluate and visualize the interactions between the two proteins.

### 2.12 Reverse transcription‐quantitative PCR (RT‐qPCR)

Using an RNA extraction kit (EZBioscience, USA), we isolated RNA from neurons and spinal cord tissues following the manufacturer's protocol and synthesized cDNA from RNA using a reverse‐transcription kit (Takara, Japan). We conducted qPCR with SYBR Green (Takara, Japan) and normalized the expression levels to GAPDH. Supplemental Table (Table S1) shows all RT‐qPCR primer sequences.

### 2.13 Plasmids and lentiviral transfection

Plasmids containing the full‐length sequence or different structural domain sequences of Flag‐tagged rat Syvn1; the full‐length sequence or different structural domain sequences of Myc‐tagged rat Stat3; HA‐tagged ubiquitin (HA‐Ub); or HA‐tagged mutated Ub (HA‐Ub mutant) were purchased from Miaolingbio (China). These plasmids were transfected into HEK 293T cells using Lipofectamine 3000.

Neurons were infected with control lentiviral shRNA (sh‐Con), lentiviral shRNA‐Syvn1 (sh‐Syvn1), sh‐Stat3, sh‐Gpx4, control overexpression lentivirus (Oe‐Con), or Syvn1 overexpression lentivirus (Oe‐Syvn1) (Obio Technology, China) to knockdown or overexpress the corresponding genes in neurons. The target sequences of the shRNAs are shown in Table S2.

### 2.14 Adeno‐associated virus (AAV) infection

pAAV‐hSyn‐Syvn1‐3 × FLAG‐P2A‐EGFP‐WPRE (AAV‐Syvn1) (Obio Technology) was used to increase the expression of Syvn1 in the SCI region in vivo, with pAAV‐hSyn‐EGFP‐P2A‐3 × FLAG‐WPRE (AAV‐Con) serving as the control vector. A vector containing shRNA targeting Stat3 [pAAV‐hSyn‐EGFP‐3 × FLAG‐miR30‐shRNA (Stat3)‐WPRE (AAV‐shStat3)] or a control vector (AAV‐shCon) was used to knock down Stat3 in the injured spinal cord region. These viral vectors were injected (titre: 6 × 10^12^ viral genomes, 10 μL) into the sheaths using a micro‐syringe immediately after SCI model construction. The target sequences of the shRNAs are shown in Table S2.

### 2.15 Haematoxylin and eosin (H&E) staining

Spinal cord tissue (approximately 1 cm long) containing the injury site was embedded in paraffin, cut into 4‐μm‐thick sections, deparaffinized, rehydrated, and then stained with haematoxylin and eosin. Finally, the sections were scanned using a pathology slide scanner (Olympus, Tokyo, Japan).

### 2.16 Western blotting analysis

We extracted total proteins from cells and tissues using RIPA lysis buffer (Beyotime, China) as described previously. SDS‐PAGE was used to separate the proteins, and then they were transferred to PVDF membranes. After blocking the membranes for 1 h at room temperature with 5% non‐fat milk, they were incubated for an additional night at 4°C with primary antibodies against Gpx4 (1:1000, Proteintech), Syvn1 (1:1000, Proteintech), Stat3 (1:1000, CST), GAPDH (1:10,000, Abcam), Flag tag (1:5000, Proteintech), Myc tag (1:5000, Proteintech), HA tag (1:5000, Proteintech), and Ub (1:1000, Proteintech). Next, the membranes were incubated for 1 h at room temperature with secondary antibodies conjugated with horseradish peroxidase against rabbit IgG (1:1000, Beyotime). A Bio‐Rad chemiluminescence gel imaging equipment (Bio‐Rad, USA) was used to detect the protein bands. ImageJ was used to quantify the band intensity.

### 2.17 Immunofluorescence staining

To stain in vitro neuronal and in vivo spinal cord tissue sections after dewaxing and antigen repair, we fixed them with 4% paraformaldehyde for 20 min, permeabilized them with 0.5% Triton X‐100 for 15 min, and then blocked for 1 h at 37°C with 5% BSA. Following that, we treated the cells for a whole night at 4°C with primary antibodies against Gpx4 (1:500, Abcam), Syvn1 (1:200, Abcam), Stat3 (1:300, CST) NeuN (1:500, Abcam), Iba‐1 (1:500, Abcam), CD31 (1:100, Abcam) and Flag tag (1:1000, Proteintech). The following day, we stained the nuclei of the cells with DAPI for 5 min at room temperature after incubating them for 1 h at 37°C with secondary antibodies (1:500, Beyotime) conjugated to either Alexa Fluor 488 or 555. A high‐resolution laser confocal microscope (Leica, Germany) was used to take the images.

### 2.18 Statistical analysis

All experimental data were independently repeated at least three times. Unpaired, two‐tailed Student's *t*‐tests were used to compare the two groups. One‐way or two‐way ANOVA was used for comparisons between more than two groups, and Tukey's post‐hoc test was used afterward. Statistics were deemed significant if *p*<0.05.

## 3 RESULTS

### 3.1 Syvn1 may be a ferroptosis‐associated protein in neurons

**FIGURE 1:**
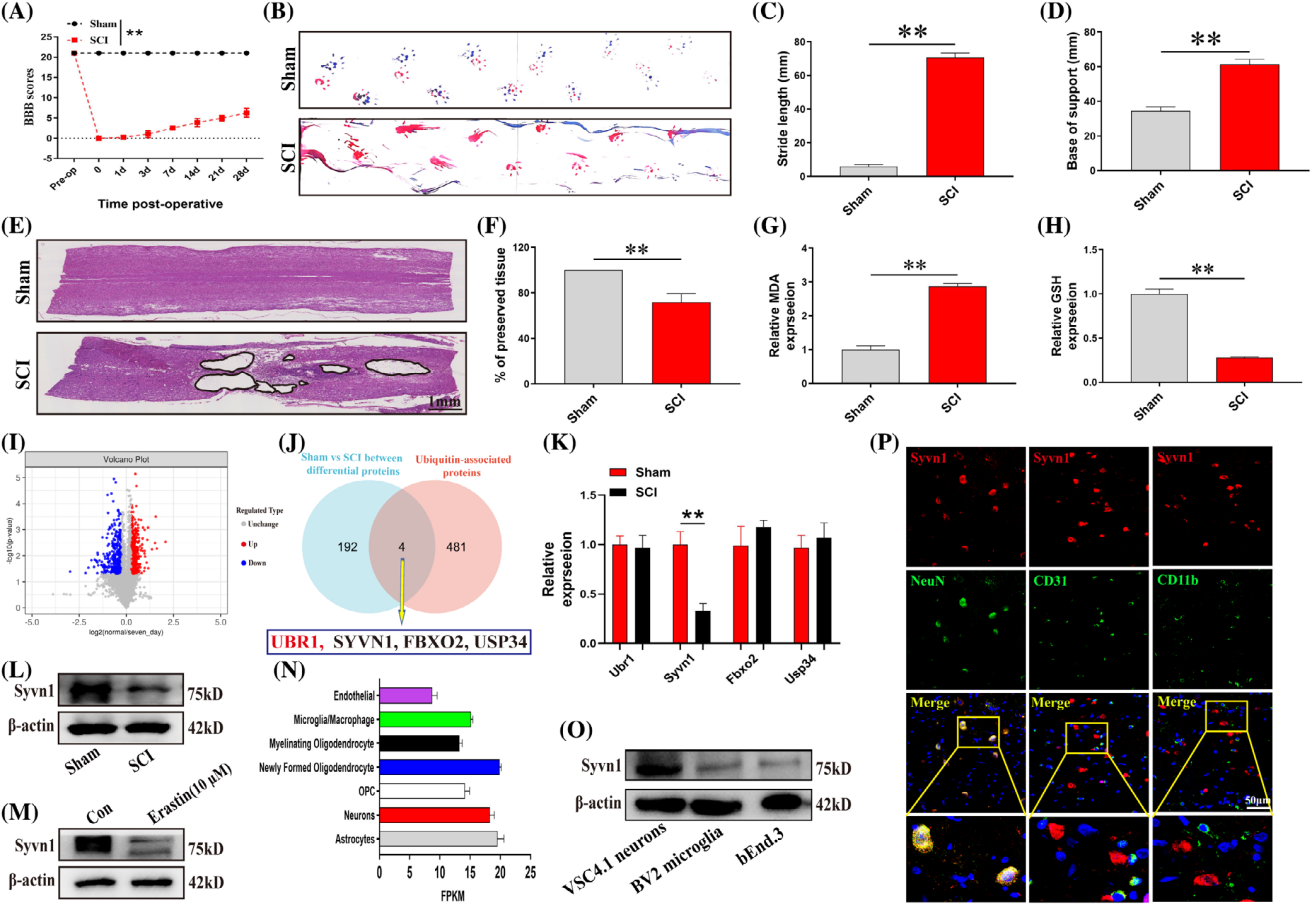
Syvn1 may play an important role in neuronal ferroptosis. (A)–(D) On day 28 after SCI, functional recovery in rats was assessed using Basso Beattie Bresnahan (BBB) scores and footprint analysis (*n* = 3). (E), (F) Haematoxylin and eosin (H&E) staining was used to assess the cavity area of the injured area. (*n* = 3). (G), (H) Seven days after SCI, the levels of glutathione (GSH) and malondialdehyde (MDA) were detected at the site of injury in each group (*n* = 3). (I) Volcano plots were used to show the relative protein expression of Sham vs SCI spinal cord tissue. Proteins with red and blue dots represent significant differential expression. (J) Venn diagram showing 4 candidate proteins generated by overlapping differential proteins in Sham versus SCI proteomics and ubiquitination‐related genes. (K) The mRNA expression levels of Ubr1, Syvn1, Fbxo2, and Usp34 in Sham and SCI spinal cord tissues were examined by RT‐qPCR. (L), (M) Detection of Syvn1 levels in spinal cord tissues and neurons by western blotting (*n* = 3). (N) Syvn1 is expressed in several cell types in the central nervous system as predicted by the Brain RNA‐seq online database (brainrnaseq.org). (O) Syvn1 expression levels in neurons, microglia, and endothelial cells were detected by western blot (*n* = 3). (P) Immunofluorescence‐based detection of Syvn1 expression levels in neurons, microglia, and endothelial cells (*n* = 3). **p<0.01.

BBB score, footprint analysis, and H&E staining results suggested that the SCI rat model was constructed successfully (Figure 1A–F; *p*<0.01). Ferroptosis has been shown to be an important pathologic mechanism in SCI.^17^ GSH depletion, Fe^2+^ accumulation, and lipid peroxidation are the key inducers of ferroptosis.^18^, ^19^ As expected, the ferroptosis phenomenon was activated after SCI (MDA upregulated; GSH downregulated; Figure 1G, H; *p*<0.01). To explore the alterations in ferroptosis markers after SCI, we performed proteomics analysis of spinal cord tissues from rats after SCI (Figure 1I). Ubiquitin ligase E3 and DUBs, as key regulatory proteins in the ubiquitin process, are also key regulatory targets in neurological diseases.^14^ Therefore, we obtained a list of 485 ubiquitin‐related genes from the E3 ligase database (https://awi.cuhk.edu.cn/~ubinet/browseE3.php) and the DUBs database (https://esbl.nhlbi.nih.gov/Databases/KSBP2/Targets/Lists/DUBs/). Overlap analysis with proteins differentially expressed in proteomics (≥1.5‐fold up‐ and down‐regulation; *p*<0.05) identified four possible ferroptosis‐associated ubiquitin proteins: Ubr1, Syvn1, Fbxo2 and Usp34 (red genes represent up‐regulation and black genes represent down‐regulation compared with the Sham group; Figure 1J). RT‐qPCR showed that the expression of Syvn1 was lower in the spinal cord tissues of SCI rats compared with the Sham group (Figure 1K; *p*<0.01). Further western blotting revealed that the protein level of Syvn1 was also down‐regulated after SCI (Figure 1L). Furthermore, a CCK‐8 assay showed that cell viability (~50%) was significantly reduced at the erastin concentration of 10 μM (Figure S1A). Interestingly, Syvn1 was also significantly reduced in elastin‐induced neuronal ferroptosis (Figure 1M). Next, Syvn1 was predicted to be highly expressed in neurons according to the online database Brain RNA‐seq (brainrnaseq.org) (Figure 1N), and Western blotting and immunofluorescence assay further confirmed that Syvn1 was mainly expressed in neurons (Figure 1O, P). These data suggest that Syvn1 is downregulated in SCI and may play an important role in neuronal ferroptosis.

Syvn1 inhibits neuronal ferroptosis and promotes function recovery in SCI rat model.

**FIGURE 2:**
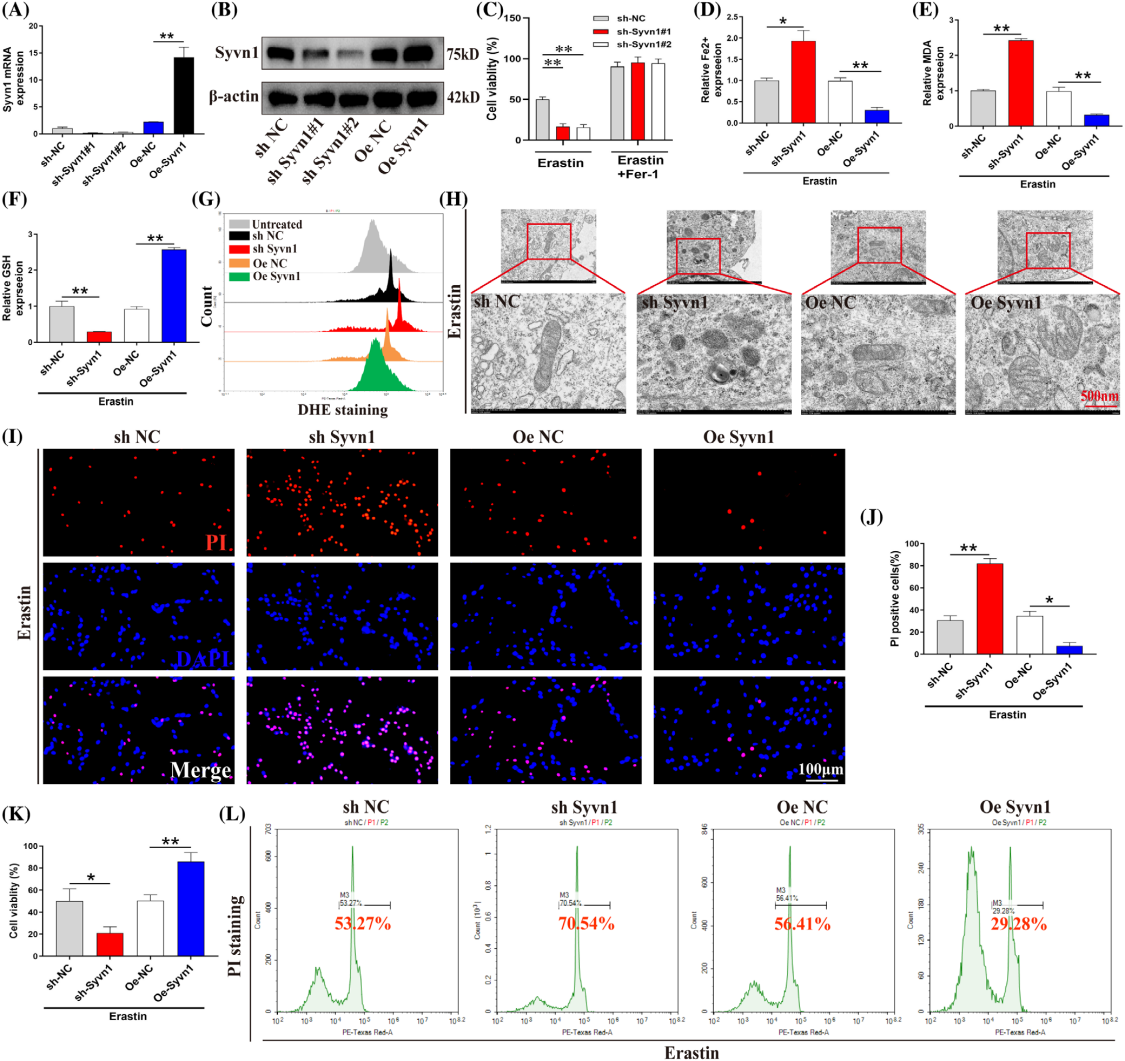
Syvn1 inhibits neuronal ferroptosis and promotes cell survival. (A), (B) VSC4.1 neurons were transfected with lentiviruses expressing sh‐Syvn1 (Syvn1 knockdown) or Oe‐Syvn1 (Syvn1 overexpression). Transfection efficiency was detected by qPCR and western blotting (*n* = 3). (C) Neurons were depleted of Syvn1 and then treated with erastin (10 μM) and Ferrostatin‐1 (Fer‐1,2 μM) for 24 h. Subsequently, neuron survival was assessed by CCK‐8 assay (*n* = 3). (D)–(F) Measurement of GSH, Fe^2+^, and MDA concentrations (*n* = 3). (G) Neurons were incubated with a DHE fluorescent probe, following which ROS levels were measured by flow cytometry. (H) Neuronal mitochondrial morphology was assessed by electron microscopy (*n* = 3). (I)–(L) Neuronal death was detected by PI staining, CCK‐8 assay, and flow cytometry (*n* = 3). *P < 0.05, **p<0.01.

Next, we generated neurons knockdowning or overexpressing Syvn1 to ascertain whether Syvn1 directly participates in ferroptosis (Figure 2A, B; *p*<0.01). First, we treated neurons with the ferroptosis inhibitor Ferrostatin‐1, and a CCK‐8 assay showed that ferrostatin‐1 treatment rescued the loss of cell viability promoted by Syvn1 knockdown (Figure 2C; *p*<0.01). Next, we found that the knockdown of Syvn1 aggravated ferroptosis‐related events (Fe^2+^, MDA, and ROS concentrations were increased; GSH levels were decreased; mitochondria displayed shrinkage, membrane thickening, and a significant reduction in the number of mitochondrial cristae in neurons); however, these results were reversed in neurons overexpressing Syvn1 (Figure 2D–H; *p*<0.05). Moreover, PI staining and a CCK‐8 assay indicated that Syvn1 enhanced neuronal viability (Figure 2I–L; *p*<0.05). These results suggested that Syvn1 improves cell survival by inhibiting neuronal ferroptosis.

**FIGURE 3:**
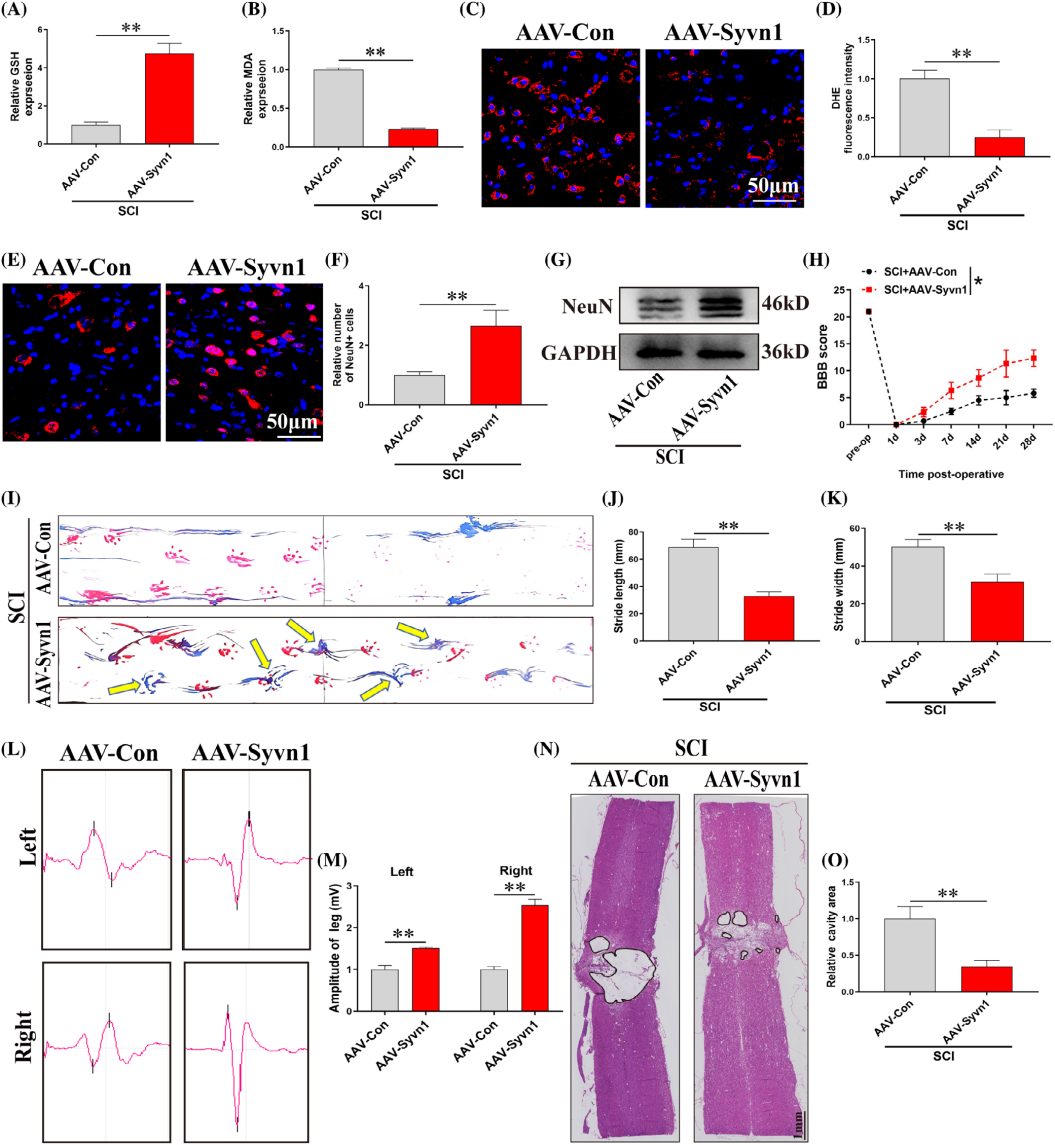
Syvn1 inhibits neuronal ferroptosis and improves functional recovery in spinal cord injury (SCI) model rats. (A), (B) Seven days after SCI, the levels of GSH and MDA were detected at the site of injury in each group (*n* = 3). (C), (D) Seven days after SCI, the levels of ROS in the injured spinal cord were quantified by DHE staining (*n* = 3). (E), (F) On day 28 after SCI, the number of NeuN‐positive neurons in the injured region of the spinal cord was quantified using immunofluorescence (*n* = 3). (G**)** On day 28 after SCI, NeuN expression in spinal cord tissues was detected by western blotting (*n* = 3). (H)–(K) On day 28 after SCI, functional recovery in rats was assessed using BBB scores and footprint analysis (*n*>3). (L), (M) Electrophysiology of rats was assessed using electromyography on day 28 after SCI (*n* = 3). (N), (O) Following H&E staining, the area of the cavity of the injured region was measured. *p<0.05, **p<0.01.

We used a neuronal‐specific AAV to overexpress Syvn1 (AAV‐Syvn1) after SCI. The results confirmed that the injection of AAV‐Syvn1 increased the expression of Syvn1 in both sham‐operated and SCI rats (Figure S2A, B), and AAV‐Syvn1 was expressed in spinal cord neurons (Figure S2C). We also found that the spinal cord region of AAV‐Syvn1‐treated rats exhibited a significant decrease in ferroptosis level (Figure 3A–D; *p*<0.01) and increased neuron numbers (Figure 3E–G; *p*<0.01) compared to the AAV‐Con group. Furthermore, assessment of motor function and pathologic staining showed that Syvn1 overexpressing rats showed a significant recovery of motor function (Figure 3H–M; *p*<0.05) and a reduction in cavity area (Figure 3N, O; *p*<0.01). Taken together, these results suggested that Syvn1 promotes function repair in rats with SCI by inhibiting neuronal ferroptosis, improving cell viability, and reducing cavity formation.

### 3.2 Syvn1 interacts with Stat3 protein

**FIGURE 4:**
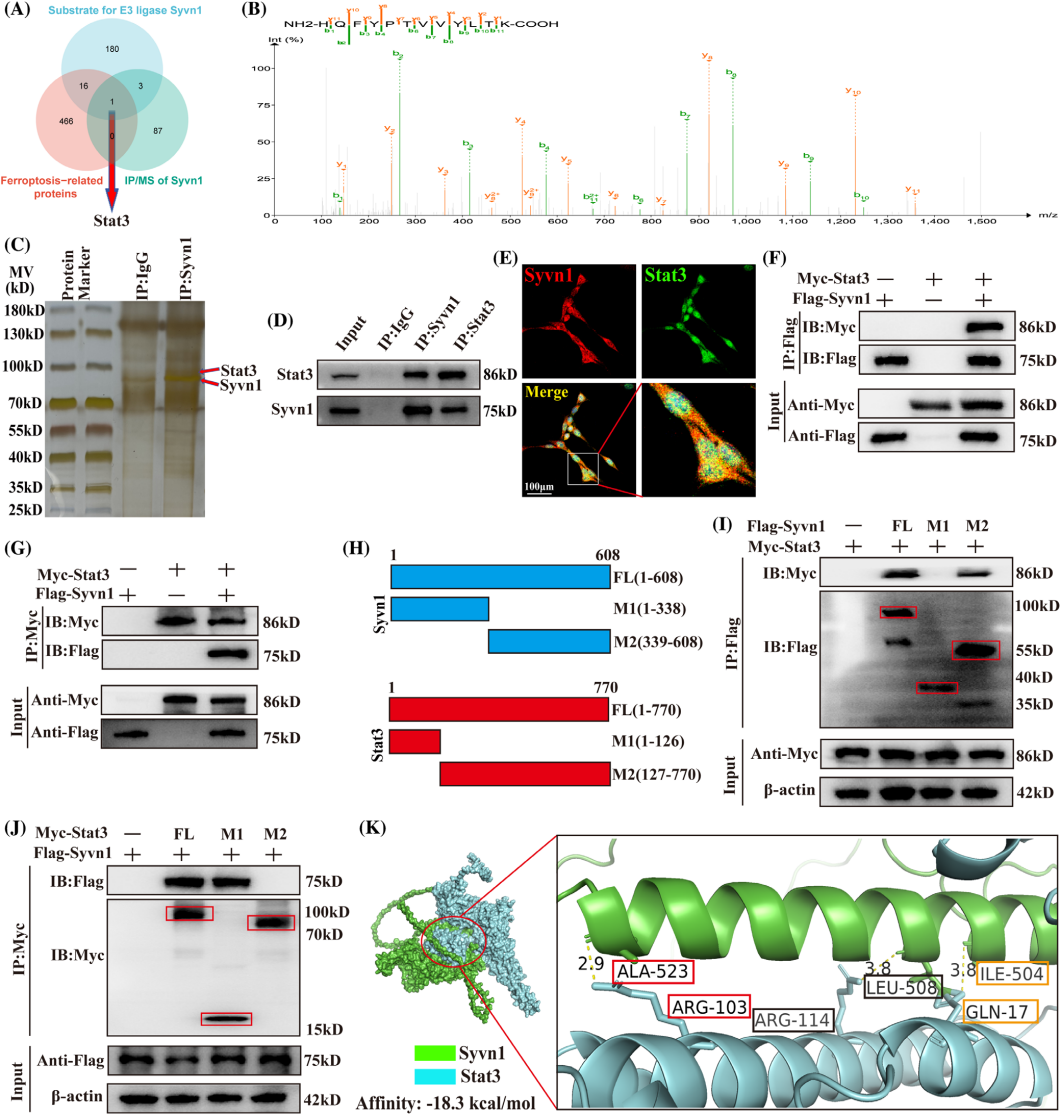
Syvn1 interacts with Stat3. (A) Interacting proteins between Syvn1's interacting substrates and ferroptosis disease‐associated genes were derived using IP/MS results of Syvn1 and bioinformatics online analysis. (B) IP/MS results indicated that Stat3 can interact with Syvn1 in VSC4.1 neurons. (C) Syvn1 co‐precipitating proteins were visualized by silver staining. (D) Co‐IP‐based detection of Syvn1 and Stat3 in VSC4.1 neurons (*n* = 3). (E) Syvn1 and Stat3 localization in neurons was assessed by immunofluorescence staining (*n* = 3). (F), (G) Flag‐tagged Syvn1 and Myc‐tagged Stat3 co‐precipitated in co‐IP experiments in HEK 293T cells (*n* = 3). (H) HEK 293T cells were transfected with full‐length or truncated fragments of Flag‐Syvn1 and Myc‐Stat3 to identify the regions involved in the binding of the two proteins. (I) Co‐IP experiments showed the binding of different Flag‐tagged Syvn1 fragments to Stat3 (*n* = 3). (J) Reverse co‐IP demonstrating the reciprocal binding of different Myc‐tagged Stat3 fragments to Syvn1. (K) Three‐dimensional modelling showing the binding of Stat3 to Syvn1 (*n* = 3).

We first performed IP/MS to identify proteins interacting with Syvn1 and used the ubiquitinating enzyme database (http://ubibrowser.bio-it.cn) to search for Syvn1 substrates, and the ferroptosis‐related diseases (http://www.zhounan.org) to search for ferroptosis‐related genes. We screened Stat3 as a ubiquitin‐modification‐associated ferroptosis regulatory protein of Syvn1 (Figure 4A, B). Silver staining also suggested that Syvn1 might bind to Stat3 (Figure 4C), while Co‐IP and immunofluorescence observed that Syvn1 and Stat3 can be significantly co‐localized in neurons (Figure 4D, E). Furthermore, Co‐IP experiments using epitope‐tagged proteins in HEK 293T cells demonstrated that Flag‐tagged Syvn1 and Myc‐tagged Stat3 also efficiently co‐precipitated (Figure 4F, G). To further investigate the specific regions through which the two proteins interact, we examined the binding domains of Syvn1 and Stat3 in HEK 293T cells using full‐length proteins and a series of truncated Flag‐tagged Syvn1 and Myc‐tagged Stat3 fragments (Figure 4H). We found that the Syvn1 fragment containing amino acids 339–608 could bind to the Stat3 fragment containing amino acids 1–126 (Figure 4I, J). Molecular docking analysis of the protein also showed that Syvn1 and Stat3 of amino acids in these intervals could interact (Figure 4K).

### 3.3 Syvn1 enhances the ubiquitination and stabilization of Stat3

**FIGURE 5:**
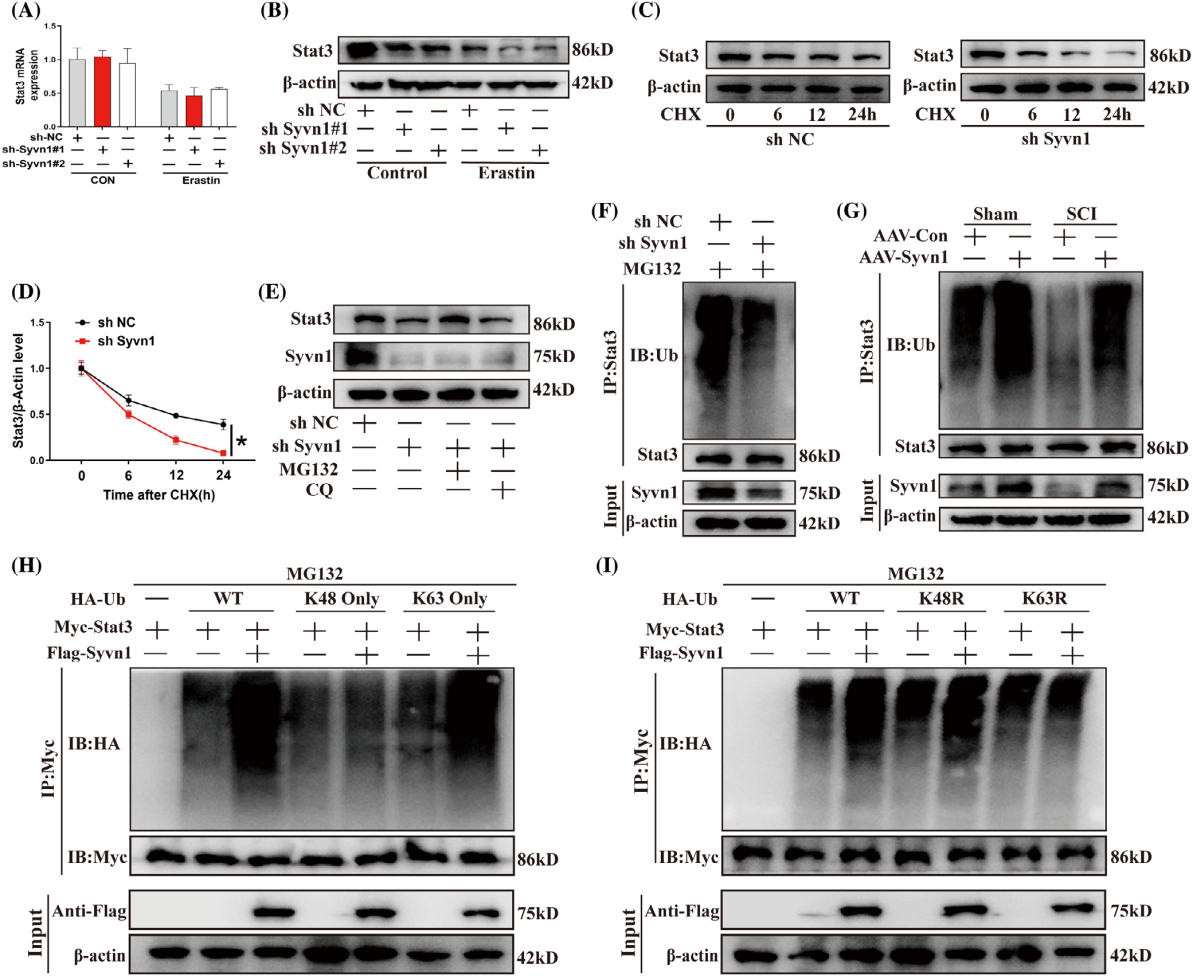
Syvn1 enhances the K63‐linked ubiquitination and stabilization of Stat3. (A), (B) VSC4.1 neurons were transfected with sh‐Syvn1#1‐ or sh‐Syvn1#2‐expressing lentiviruses and then treated or not with erastin (10 μM) for 24 h. The level of Stat3 was detected by qPCR and western blotting (*n* = 3). (C), (D) sh‐NC‐ or sh‐Syvn1‐expressing neurons were treated with cycloheximide (CHX, 10 μg/mL) for different durations (0, 6, 12, 24 h) following which Stat3 protein levels were quantified by western blotting (*n* = 3). (E) The levels of Stat3 and Syvn1 in Syvn1‐knockdown neurons treated with MG132 (20 μM) or chloroquine (CQ, 20 μM) for 12 h were quantified by western blotting (*n* = 3). (F) Stat3 ubiquitination and Syvn1 protein levels in Syvn1‐knockdown neurons were assessed by western blot (*n* = 3). (G) In rats injected with AAV‐Con or AAV‐Syvn1, ubiquitination of Stat3 and protein levels of Syvn1 were observed by western blotting (*n* = 3). (H), (I) HEK 293T cells were co‐transfected with Flag‐Syvn1 and Myc‐Stat31, and then also with HA‐Ub‐WT, HA‐Ub‐K48‐only, HA‐Ub‐K63‐only, HA‐Ub‐K48R, or HA‐Ub‐K63R plasmids, following which Stat3 ubiquitination levels were analysed (*n* = 3). *p<0.05.

We found that the knockdown of Syvn1 reduced the protein levels of Stat3 but did not influence its transcription (Figure 5A, B). Moreover, we blocked Stat3 synthesis using cycloheximide and observed that Syvn1 knockdown enhanced Stat3 protein degradation (Figure 5C, D; *p*<0.05). We further treated neurons with the proteasome inhibitor MG132 and the autophagy inhibitor chloroquine to identify the pathway involved in Stat3 degradation and found that MG132 reversed the Syvn1 knockdown‐mediated decrease in Stat3 protein levels (Figure 5E). This suggested that Syvn1 modulates Stat3 protein stability through the ubiquitin–proteasome pathway. Next, we examined the effect of Syvn1 on the ubiquitination level of Stat3 protein and found that the knockdown of Syvn1 significantly reduced the ubiquitination level of Stat3 in neurons (Figure 5F). Moreover, we observed that the ubiquitination levels of Stat3 was higher in rats injected with AAV‐Syvn1 than in Sham‐AAV‐Con or SCI‐AAV‐Con animals (Figure 5G). The ubiquitin molecule has seven lysine residues, all of which can participate in the synthesis of polyubiquitin chains.^20^ The K48‐conjugated ubiquitin chain mainly mediates protein degradation,^21^ whereas the K63 ubiquitin chain is primarily involved in the regulation of protein function and the maintenance of protein stability.^22^ To investigate the effect of Syvn1 on Stat3 polyubiquitination, we transfected HEK 293 T cells with plasmids expressing HA‐Ub‐WT, HA‐Ub‐K48, or HA‐Ub‐K63 and found that the overexpression of Syvn1 significantly increased the ubiquitination level of Stat3 in cells transfected with HA‐Ub‐WT or HA‐Ub‐K63 but not HA‐Ub‐K48 (Figure 5H). Subsequently, we transfected HEK 293T cells with plasmids expressing HA‐Ub‐WT, HA‐Ub‐K48R (lysine 48 was replaced with an arginine), or HA‐Ub‐K63R (lysine 63 replaced with an arginine) and found that the transfection of HA‐Ub‐K63R abolished the Syvn1 overexpression‐mediated increase in Stat3 ubiquitination levels (Figure 5I). These results suggested that Syvn1 promotes Stat3 protein stability through K63 ubiquitin chain‐mediated ubiquitination.

### 3.4 Syvn1 inhibits neuronal ferroptosis in a Stat3‐dependent manner

**FIGURE 6:**
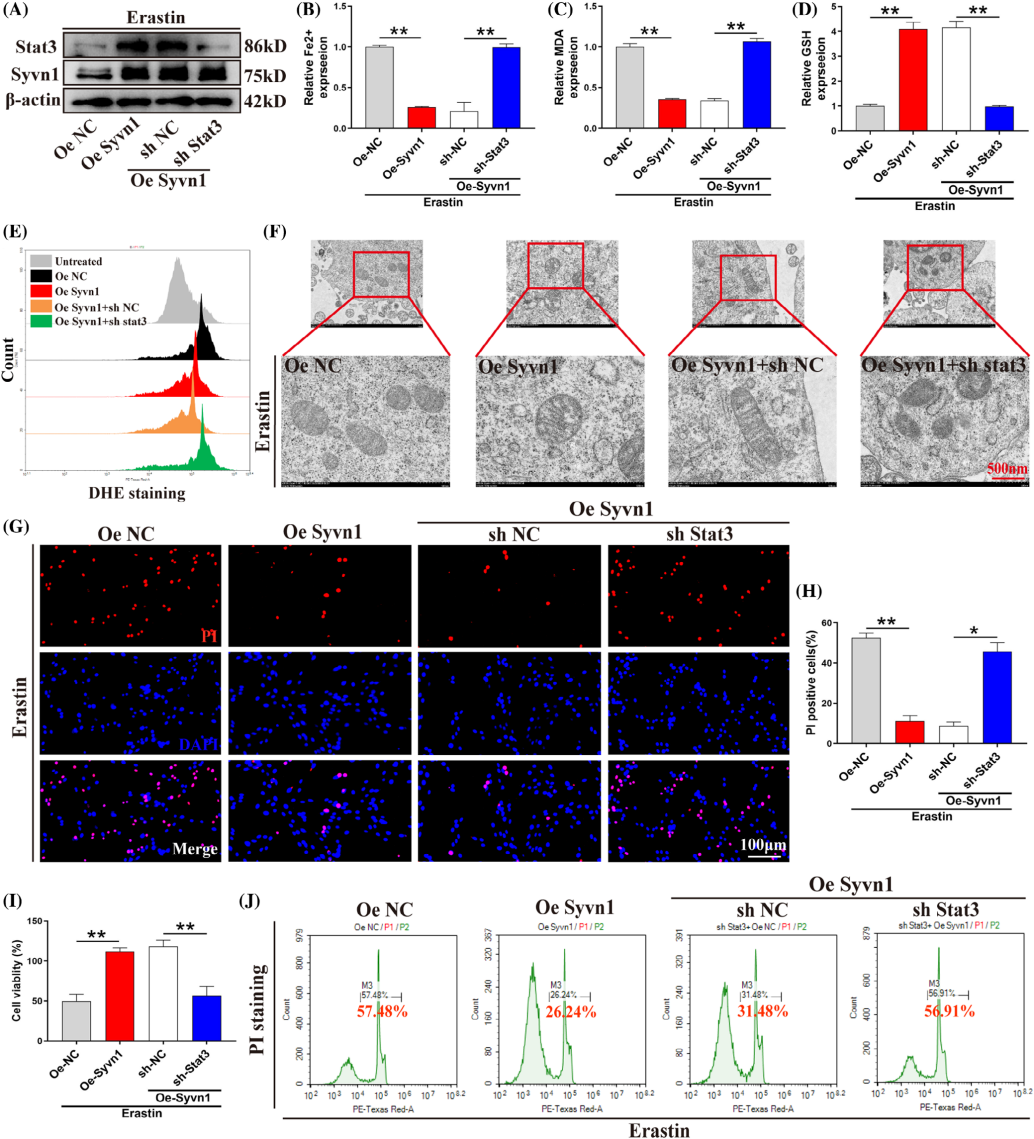
Syvn1 inhibits neuronal ferroptosis in a Stat3‐dependent manner. (A) Oe‐Syvn1 (Syvn1 overexpression) and sh‐Stat3 (Stat3 knockdown) + Oe‐Syvn1 lentiviruses were transfected into VSC4.1 neurons, respectively, and the transfection efficiency was detected by western blotting (*n* = 3). (B)–(D) Fe^2+^, GSH, and MDA concentrations were examined using the respective kits (*n* = 3). (E) Treated neurons were incubated with a DHE fluorescent probe after which ROS levels were assessed by flow cytometry (*n* = 3). (F) Electron microscopic observation of mitochondrial morphology in neurons (*n* = 3). (G)–(J) Cell viability was assessed by PI staining, CCK‐8 assay, and flow cytometry (*n* = 3). *p<0.05, **p<0.01.

To examine the function of Stat3 in the regulation of ferroptosis downstream of Syvn1, we generated VSC 4.1 neurons that overexpressed Syvn1 or anaplerotically knocked down Stat3 (Figure 6A). We observed that Syvn1 overexpression attenuated ferroptosis and promoted cell survival in neurons, while Stat3 knockdown in Syvn1‐overexpressing neurons reversed these effects (Figure 6B–J; *p*<0.05). We further evaluated whether Syvn1 relies on Stat3 to regulate neuronal ferroptosis in primary cortical neurons. As expected, Syvn1 inhibited ferroptosis (decreased MDA content and Fe^2+^ concentration) in primary neurons, and the antiferroptotic effect of Syvn1 was reversed upon knockdown of Stat3 in primary neurons (Figure S3A–C; *p*<0.01). These findings implied that Stat3 is crucial for the Syvn1‐mediated suppression of neuronal ferroptosis.

### 3.5 Syvn1 inhibits neuronal ferroptosis through the Stat3/Gpx4 pathway

**FIGURE 7:**
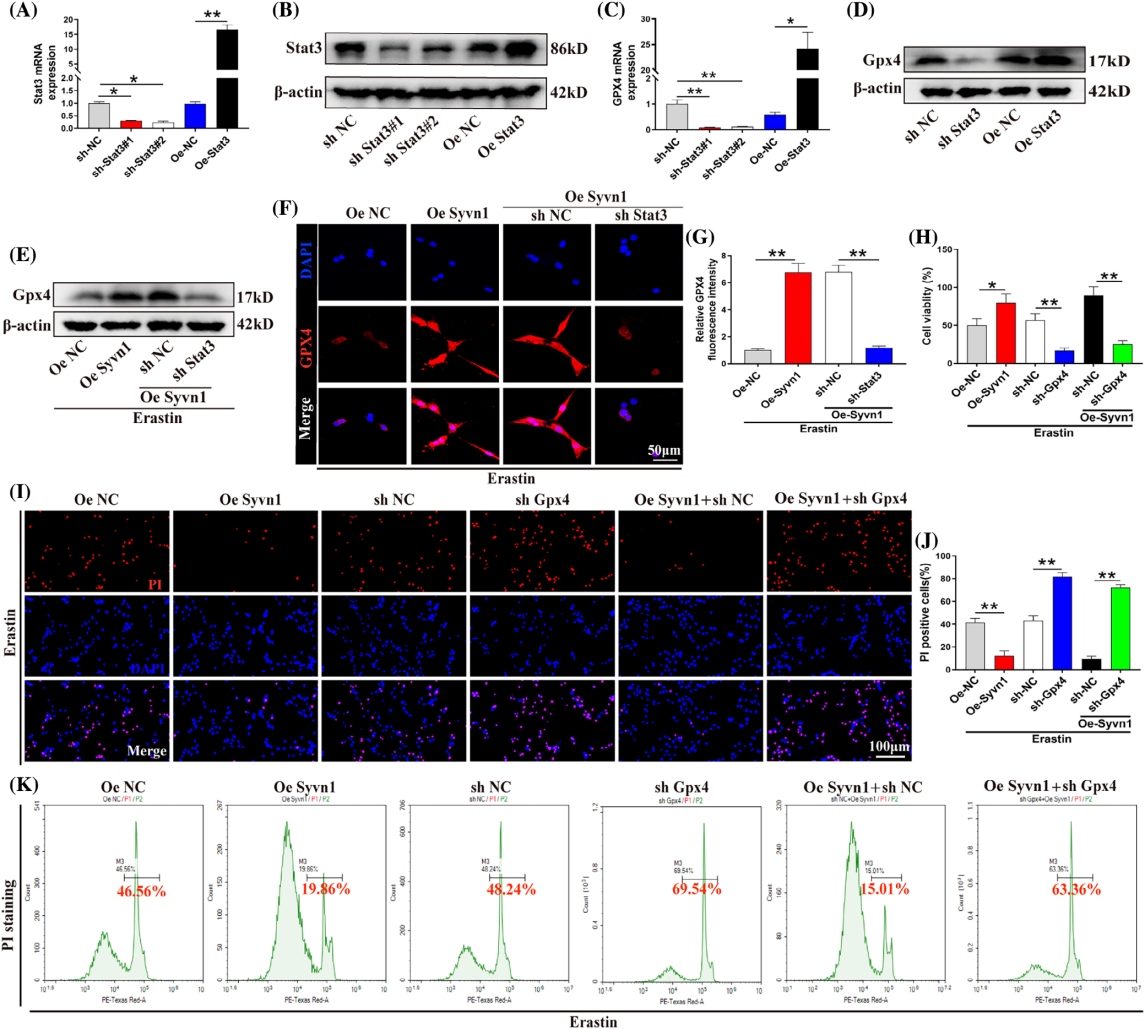
Syvn1 inhibits neuronal ferroptosis through the Stat3/Gpx4 pathway. (A), (B) sh‐Stat3#1, sh‐Stat3#2 (Stat3 knockdown), and Oe‐Stat3 (Stat3 overexpression) lentiviruses were transfected into VSC4.1 neurons and their transfection efficiencies were analysed by qPCR and western blotting (*n* = 3). (C), (D) The effect of Stat3 knockdown and overexpression on Gpx4 transcript and protein levels was detected by qPCR and western blot, respectively (*n* = 3). (E) The protein level of Gpx4 was measured by western blotting (*n* = 3). (F), (G) Immunofluorescence detection and quantification of Gpx4 fluorescence intensity (*n* = 3). (H)–(K) Cell viability was assessed by PI staining, CCK‐8 assay, and flow cytometry (*n* = 3). *p<0.05, **p<0.01.

Stat3 binds to the promoter region of Gpx4 and promotes its transcription and inhibits ferroptosis in pancreatic cancer cells^23^; however, whether Stat3 can regulate Gpx4 expression by promoting its transcription in neurons is not known. We found that Stat3 knockdown downregulated the mRNA and protein expression levels of Gpx4 in neurons, whereas Stat3 overexpression exerted the opposite effect (Figure 7A–D; *p*<0.05). Additionally, Syvn1 overexpression led to the upregulation of the protein expression level of Gpx4 in neurons, while Stat3 knockdown abrogated this effect of Syvn1 (Figure 7E–G; *p*<0.01). To further explore the role of Gpx4 in the Syvn1‐mediated regulation of ferroptosis in neurons, we generated Gpx4‐knockdown neurons (Figure S4A) and subsequently observed that Gpx4 knockdown reversed the inhibitory effect of Syvn1 on neuronal death (Figure 7H–K; *p*<0.05). These results suggested that Syvn1 inhibits neuronal ferroptosis by activating the Stat3/Gpx4 pathway.

### 3.6 Syvn1 attenuates neuronal ferroptosis and improves functional recovery in a SCI rat model through a Stat3‐dependent manner

**FIGURE 8:**
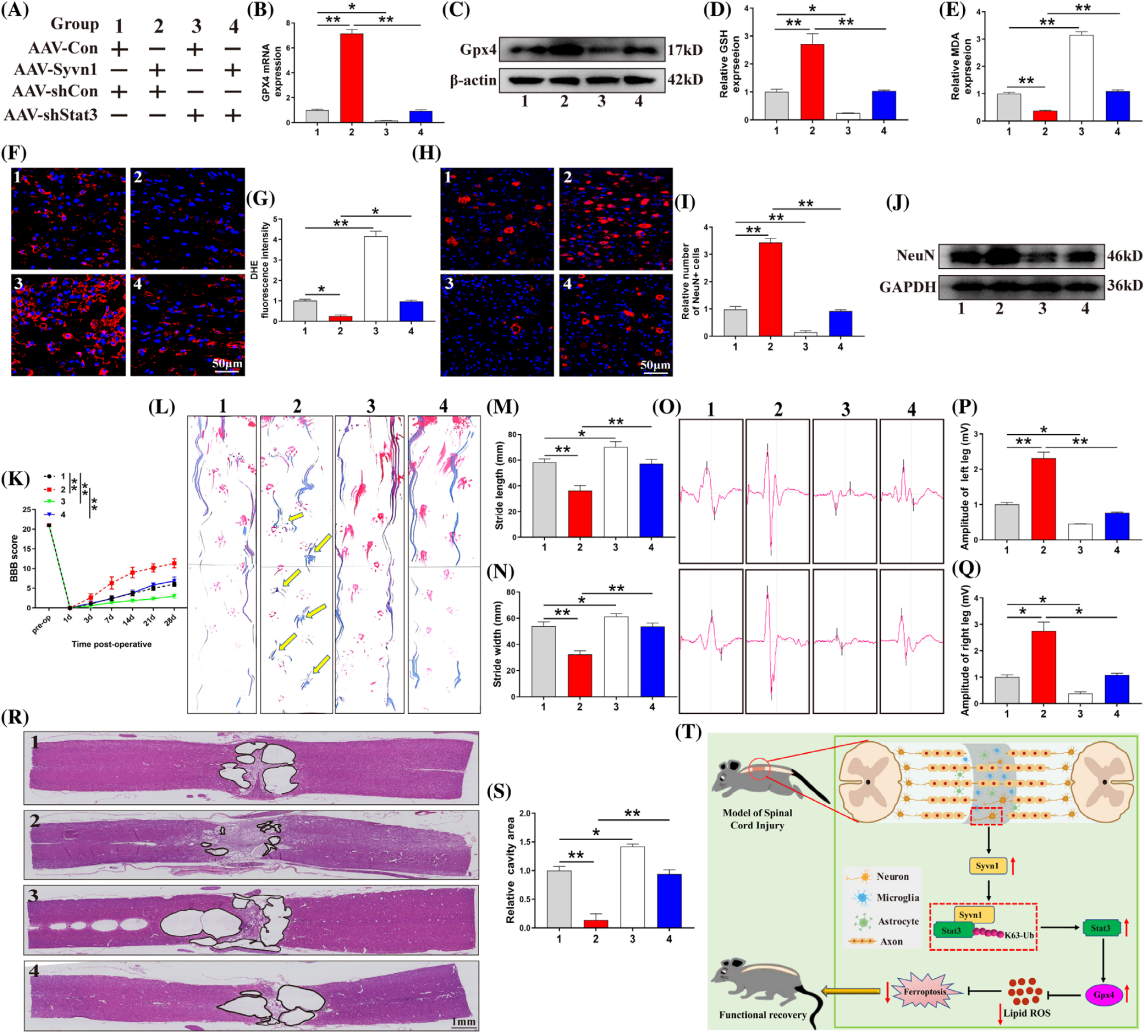
Syvn1 attenuates neuronal ferroptosis and facilitates functional recovery in SCI rats via Stat3. (A) Detailed information of each group is listed in this part of the experiment. (B), (C) Gpx4 levels were detected by qPCR and western blotting 7 days post‐SCI (*n* = 3). (D), (E) GSH, and MDA levels in the injury area were detected in each group of 7 days post‐SCI (*n* = 3). (F), (G) On day 7 after SCI, the levels of ROS in the injured spinal cord were quantified using a DHE staining kit (*n* = 3). (H), (I) On day 28 after SCI, the number of NeuN‐positive neurons in the spinal cord injury area was detected by immunofluorescence assay (*n* = 3). (J) On day 28 after SCI, the protein levels of NeuN in damaged tissues were assessed by western blotting (*n* = 3). (K)–(N) On day 28 after SCI, the extent of functional recovery in rats was assessed using BBB scores and footprint analysis (*n*>3). (O)–(Q) Electrophysiological assessment of rats was undertaken on day 28 after SCI using electromyography (*n* = 3). (R), (S) On day 28 after SCI, the area of the cavity of the injured region was measured following H&E staining (*n* = 3). (T) Syvn1 targets Stat3 to regulate its ubiquitination and stability, thereby modulating neuronal ferroptosis after SCI. *p<0.05, **p<0.01.

We first sought to identify the optimal site for in vivo injection of AAV‐shStat3 (Figure S4B). Next, we intrathecally injected different AAV into the rat spinal cord to respectively overexpress Syvn1 or knockdown Stat3 in neurons (Figure 8A). Consistent with the in vitro results, Syvn1 overexpression increased Gpx4 levels in rats after SCI, an effect that was reversed by Stat3 knockdown (Figure 8B, C; *p*<0.05). Furthermore, Syvn1 overexpression significantly attenuated ferroptosis‐related events in SCI model rats (Figure 8D–G; *p*<0.05), improved neuronal survival (Figure 8H–J; *p*<0.01), reduced the cavity area in injured regions, and promoted functional recovery. Interestingly, Stat3 knockdown reversed the anti‐ferroptotic effect of Syvn1 and impaired functional recovery (Figure 8K–S; *p*<0.05). These results suggested that Syvn1 inhibits neuronal ferroptosis and promotes functional recovery in rats after SCI in a Stat3‐dependent manner (Figure 8T).

## 4 DISCUSSION

Our results demonstrated that neuronal ferroptosis is a critical exacerbating factor for secondary injury after SCI.^7^, ^24^ Thus, elucidating the molecular mechanisms of neuronal ferroptosis is essential to facilitate SCI repair studies.^25^ In this study, we found that the E3 ubiquitin ligase Syvn1, ferroptosis‐associated protein, attenuates neuronal ferroptosis and improves functional recovery in SCI model rats. Mechanistically, Syvn1 directly targets and binds to Stat3 and maintains its protein stability, which leads to an increase in the transcription and expression level of Gpx4 and attenuates neuronal ferroptosis. Therefore, the discovery of the Syvn1‐mediated Stat3/Gpx4 pathway provides new insights into neuronal ferroptosis.

Syvn1 is involved in the regulation of a variety of biological processes, such as neural development,^26^, ^27^ and apoptosis^28^, ^29^ via recognizing and binding to specific substrate proteins and affecting their ubiquitination. An increasing number of studies have reported that Syvn1 can influence cell survival by regulating the ubiquitination of proteins involved in ferroptosis. For instance, Guo et al. found that Syvn1 inhibits ferroptosis by inducing HMGB1 ubiquitination and subsequent degradation.^30^ Additionally, Syvn1 mediates ferroptosis and cell death in blast cancer cells through the ubiquitination and degradation of ETS1.^31^ In this study, we confirmed the presence of ferroptosis after SCI, and further proteomics and experimental validation showed that Syvn1 is downregulated after SCI and is predominantly expressed in neurons. These results suggest that Syvn1 may be a ferroptosis‐related protein in neurons. However, neuronal ferroptosis after SCI is a complex process, and understanding the molecular mechanisms involved requires more in‐depth studies.

Ferroptosis is a form of programmed cell death induced by an imbalance in intracellular iron metabolism, which leads to the excessive accumulation of lipid peroxides.^32^, ^33^ The attenuated ferroptosis has been associated with functional recovery in SCI.^34^ In line with the previous findings, we showed that Syvn1 alleviated neuronal ferroptosis and improved cell survival in the injured spinal cord. We next explored the mechanism by which Syvn1 inhibits neuronal ferroptosis and identified Stat3 as a substrate and interacting partner of Syvn1 in neurons. K48‐linked ubiquitin chains are mainly involved in protein degradation,^35^ while K63‐linked ones mainly participate in the modulation of protein function and stability.^21^, ^36^ To investigate whether the ubiquitination of Stat3 by Syvn1 leads to Stat3 stabilization or degradation, we performed ubiquitination assays and found that Syvn1 enhanced Stat3 protein stability through K63‐linked ubiquitination.

As a classical transcription factor, Stat3 regulates the expression of a variety of genes in response to cellular stimuli and plays a crucial role in diverse biological processes.^37^ Studies have shown that Stat3 binds to the promoter region of Gpx4 and activates its transcription, thereby inhibiting ferroptosis in pancreatic cancer cells.^23^ Gpx4 is a central ferroptosis regulator and a key scavenger of lipid peroxides in the cell.^38^ The activity of Gpx4 is reduced during ferroptosis, resulting in the generation of lipid peroxides and ROS, which ultimately cause cell death.^39^, ^40^ Although it has been demonstrated that Stat3 can induce the transcription of Gpx4, it is well known that the regulation of gene transcription varies according to cell type and disease state, and it was unclear whether Stat3 activates Gpx4 expression in neurons. Here, we found that Stat3 knockdown reduced the protein level of Gpx4 in neurons, suggesting that Stat3 may have a role in regulating the level of Gpx4 in these cells. Overexpressing Syvn1 increased the levels of both Stat3 and Gpx4 in neurons and enhanced neuronal survival, whereas Stat3 knockdown resulted in the opposite effects. Moreover, the knockdown of Gpx4 also abolished the protective effect of Syvn1 on neuronal death. These results indicated that Syvn1 suppresses neuronal ferroptosis by activating the Stat3/Gpx4 pathway.

## 5 CONCLUSIONS

We identified a ferroptosis‐associated protein, Syvn1, which inhibits neuronal ferroptosis after SCI by targeting Stat3 and mediating Gpx4 expression, thereby promoting functional recovery. Mechanistically, we demonstrated that the E3 ligase Syvn1 interacts with its novel substrate Stat3 and enhances K63‐linked polyubiquitin chains to promote the ubiquitination level and stability of Stat3, leading to an increase in the level of Stat3‐dependent Gpx4. Whereas Gpx4 acts as a scavenger of lipid peroxides, activation of its expression is an important means of inhibiting neuronal ferroptosis. Our findings emphasize that the Syvn1‐mediated Stat3/Gpx4 axis is involved in the regulation of neuronal ferroptosis and ameliorates neuronal death after SCI. The targeted enhancement of the interaction between Syvn1 and Stat3 may be a potentially effective strategy for the treatment of SCI.

## AUTHOR CONTRIBUTIONS

Shining Xiao and Yu Zhang performed most of the experiments and wrote the manuscript. Shijiang Wang participated in most of the experiments. Jiaming Liu supervised this study. Fan Dan and Feng Yang participated in cell culture and animal modelling. Shue Hong helped with animal electromyography experiments. Ning Liu, Yujia Zeng, and Ke Huang participated in animal modelling. Xinsheng Xie and Yanxin Zhong participated in the postoperative care of the animals. Zhili Liu supervised and funded this study. All authors approved the final version of the manuscript.

## FUNDING INFORMATION

This study was supported by the Natural Science Foundation of Jiangxi Province (No. 20232ACB206023) and the “Double Thousand Plan” of Jiangxi Province.

## CONFLICT OF INTEREST STATEMENT

The authors declare no competing interests.

## Supporting information


**Data S1:** Supporting Information

**Data S2:** Ferroptosis test in Figure S3.

## Data Availability

The majority of the datasets for this study are included in this article and the supplementary material. Original data are available on reasonable request.
