# Supplementary material for: The Syvn1 inhibits neuronal cell ferroptosis by activating Stat3/Gpx4 axis in rat with spinal cord injury
Source: Cell Prolif. 2024 May 27;57(10):e13658. doi: 10.1111/cpr.13658 (PMC11471452; doi:10.1111/cpr.13658)
Supplement: Supplementary file 1 — Data S1: Supporting Information [file CPR-57-e13658-s001.docx]

**The Syvn1 inhibits neuronal cell ferroptosis by activating Stat3 /Gpx4 axis in rat with spinal cord injury**

**Supplementary Figure**

**
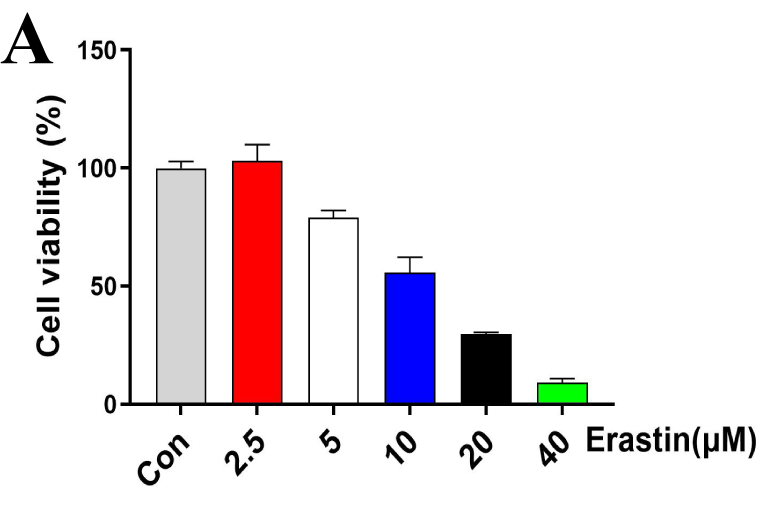
**

**Fig S1 Erastin induces neuronal cell death. A** CCK8 detects the effect of Erastin on the activity of VSC4.1 neurons after stimulation with Erastin for 24 h (*n* = 3).


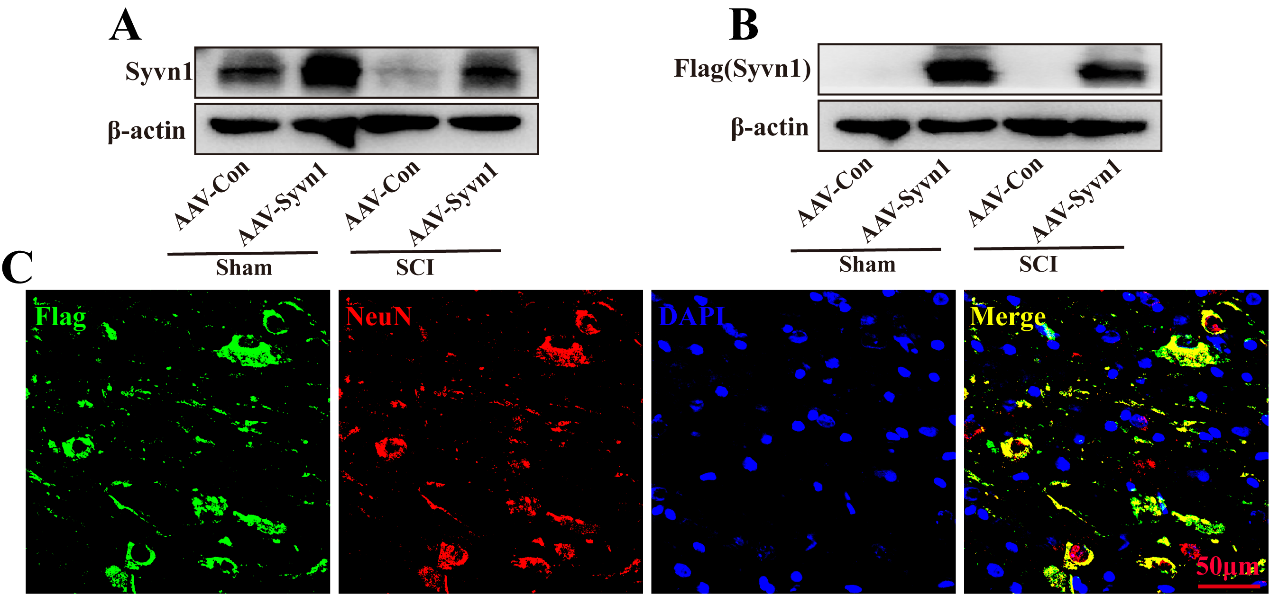


**Fig S2 Transfection efficiency of AAV-Syvn1.** **A, B** 7 days after SCI, Western blotting was uesd to test the level of Syvn1 and Syvn1(Flag) in spinal cord tissues (*n* = 3). **C.** 7 days after SCI, immunofluorescence was performed to identify the infection efficiency of AAV-Syvn1(Flag) in neurons (*n* = 3).


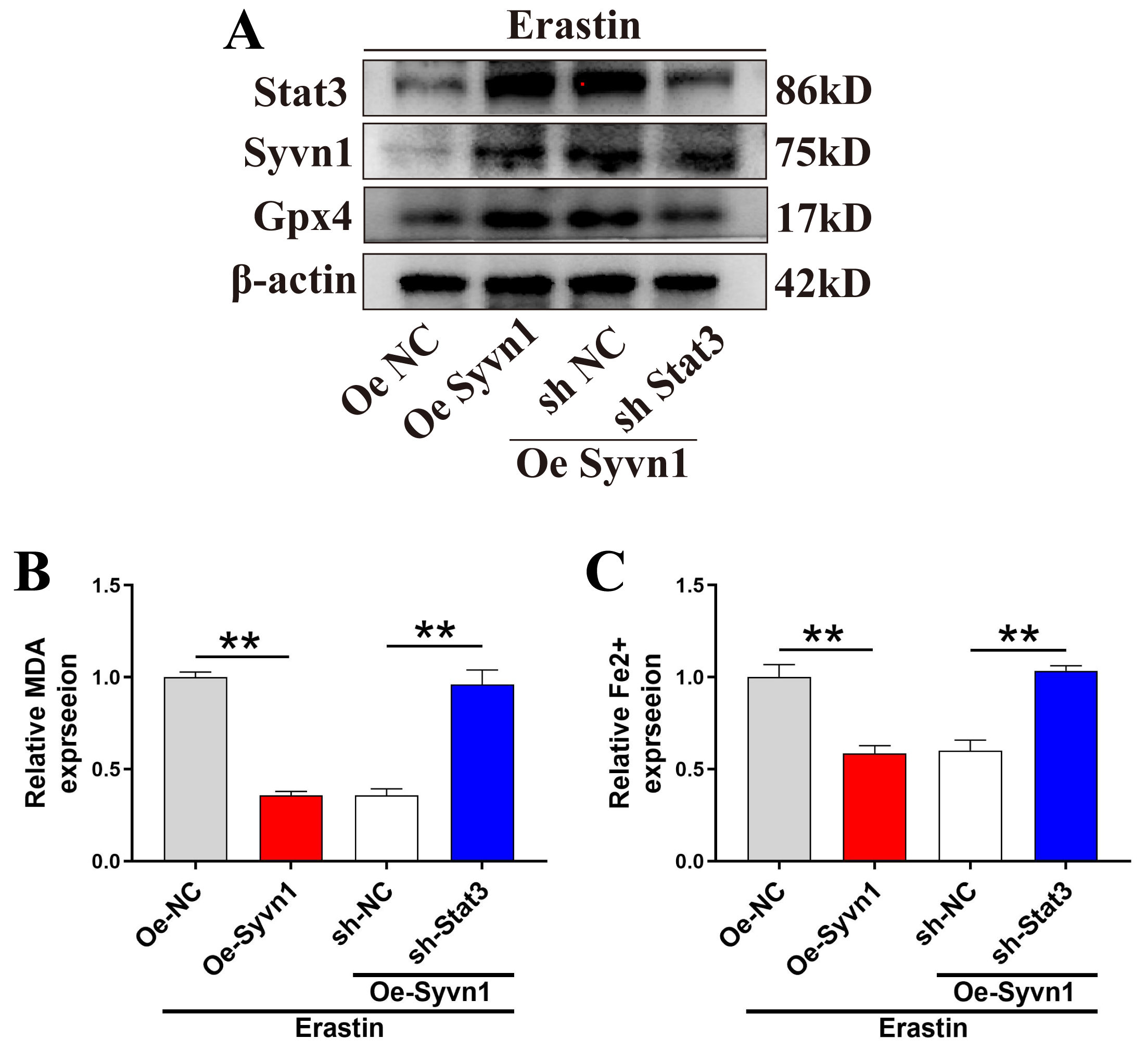


**Fig S3 Syvn1 inhibits ferroptosis in primary cortical neurons via Stat3. A** Western blotting to detect the expression levels of Syvn1, Stat3 and Gpx4 in primary cortical neurons. **B, C** MDA and Fe^2+^ concentrations were examined using the respective kits.


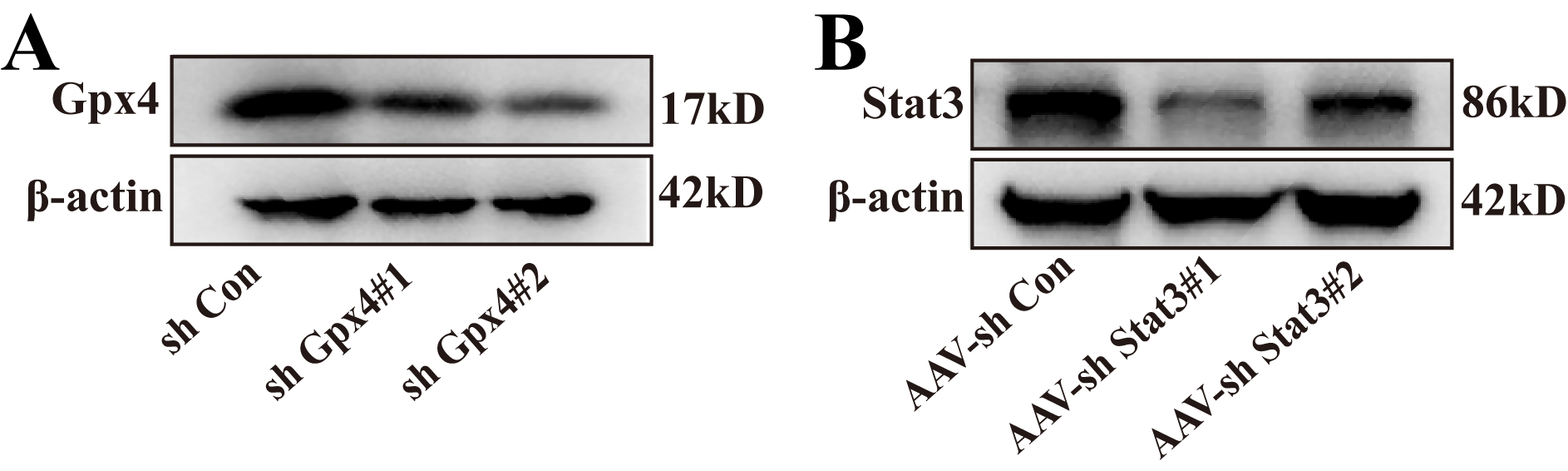


**Fig S4 Transfection efficiency of the virus. A** Western blotting to test the level of Gpx4 in neurons (*n* = 3). **B** 7 days after SCI, Western blotting to detect the level of Stat3 in spinal cord tissues (*n* = 3).

**Supplementary Table**


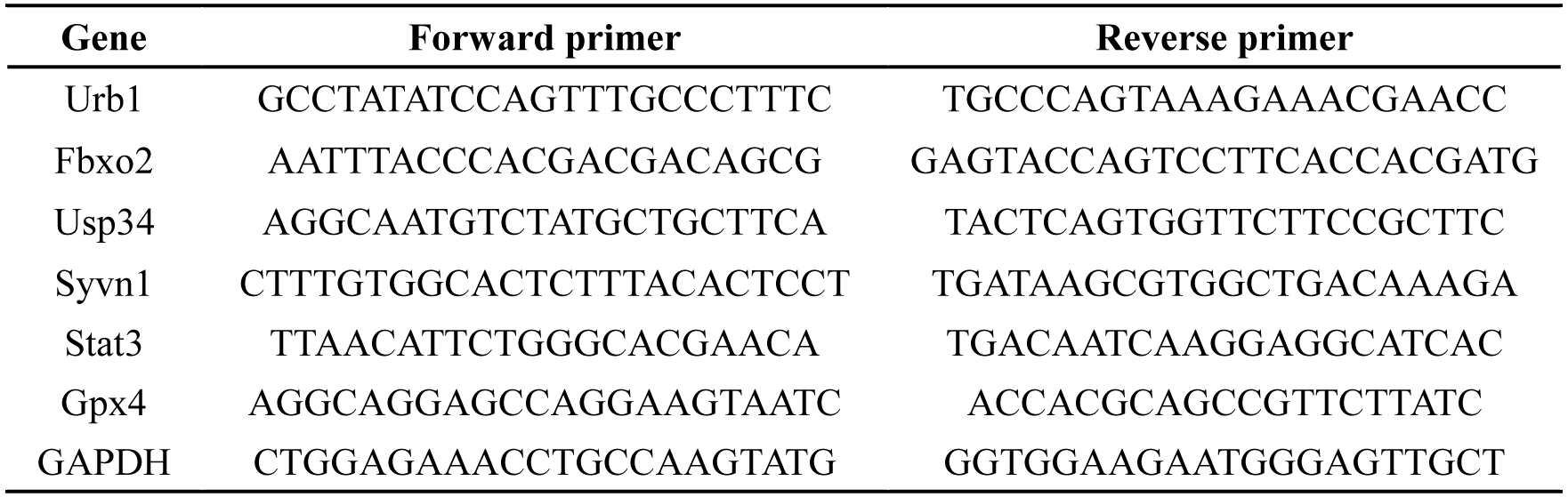


**Table S1 Primers for qRT-PCR analysis.**

**
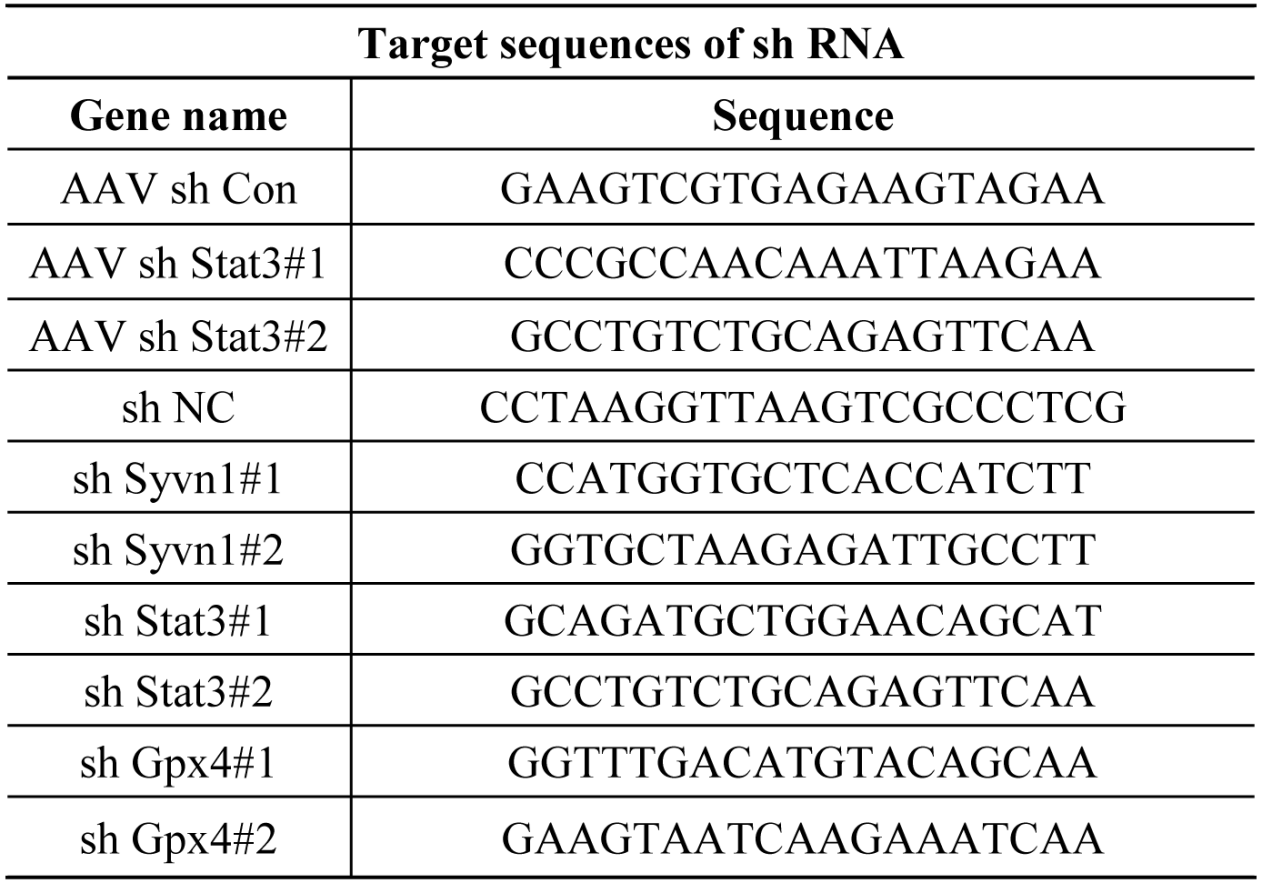
**

**Table S2 Target sequences of sh-RNA.**
