# Supplementary figures and images for: The Syvn1 inhibits neuronal cell ferroptosis by activating Stat3/Gpx4 axis in rat with spinal cord injury
Source: Cell Prolif. 2024 May 27;57(10):e13658. doi: 10.1111/cpr.13658 (PMC11471452; doi:10.1111/cpr.13658)

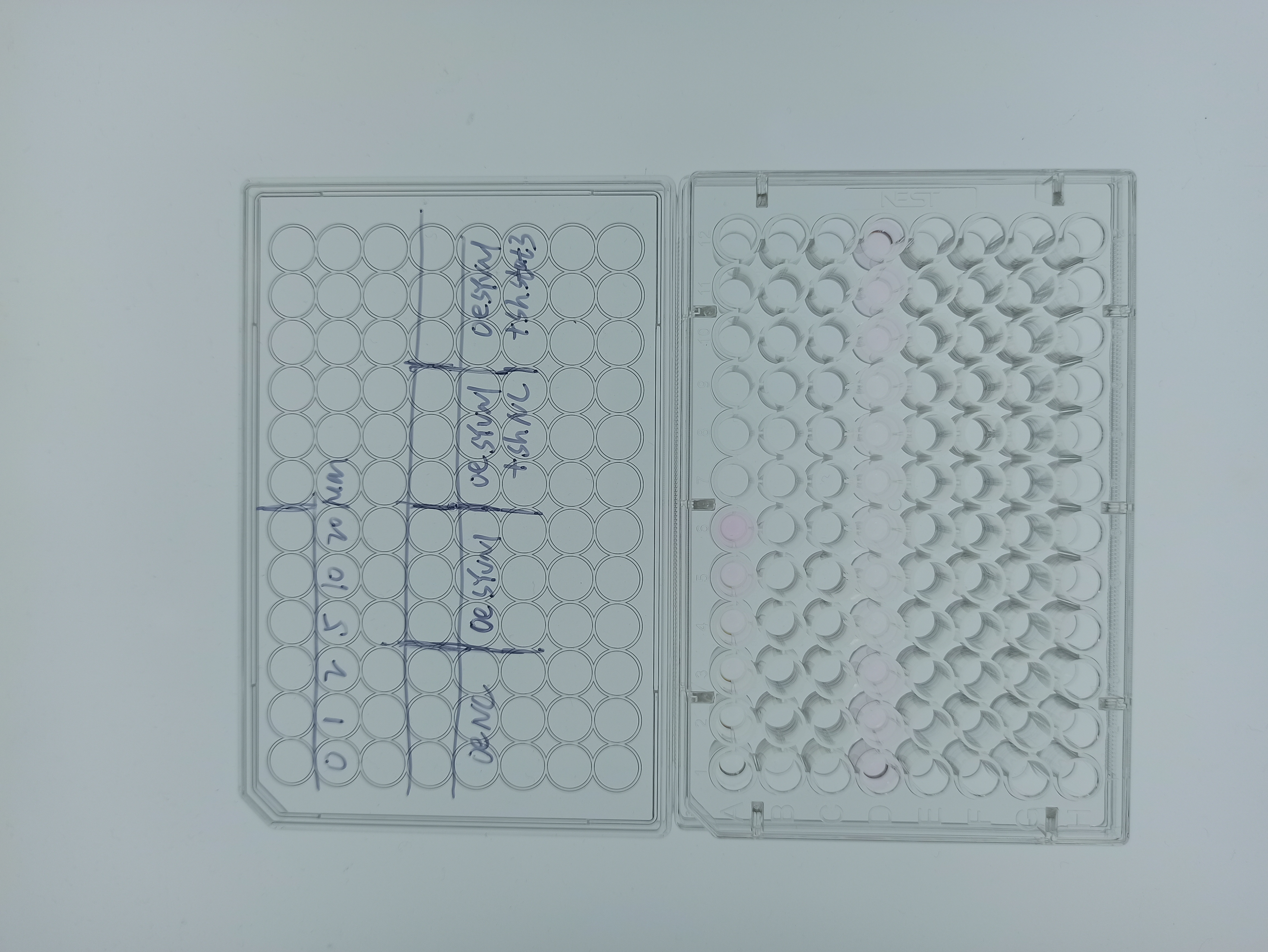

Supplement: Supplementary file 2 — Data S2: Ferroptosis test in Figure S3. [file CPR-57-e13658-s002.zip › Ferroptosis test in Fig S3/Fig S3B MDA Test/MDA blank (2).jpg]

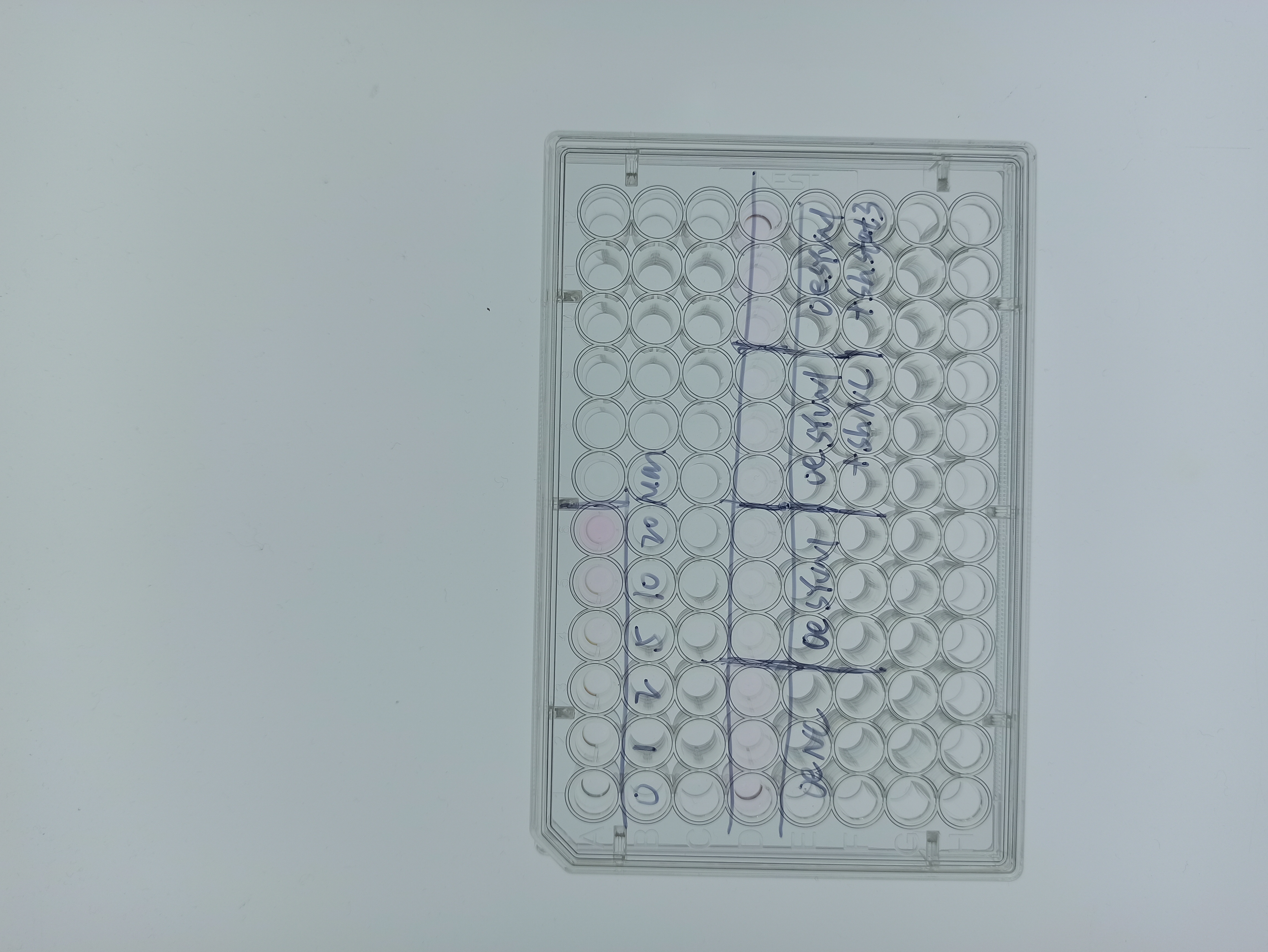

Supplement: Supplementary file 2 — Data S2: Ferroptosis test in Figure S3. [file CPR-57-e13658-s002.zip › Ferroptosis test in Fig S3/Fig S3B MDA Test/MDA blank.jpg]

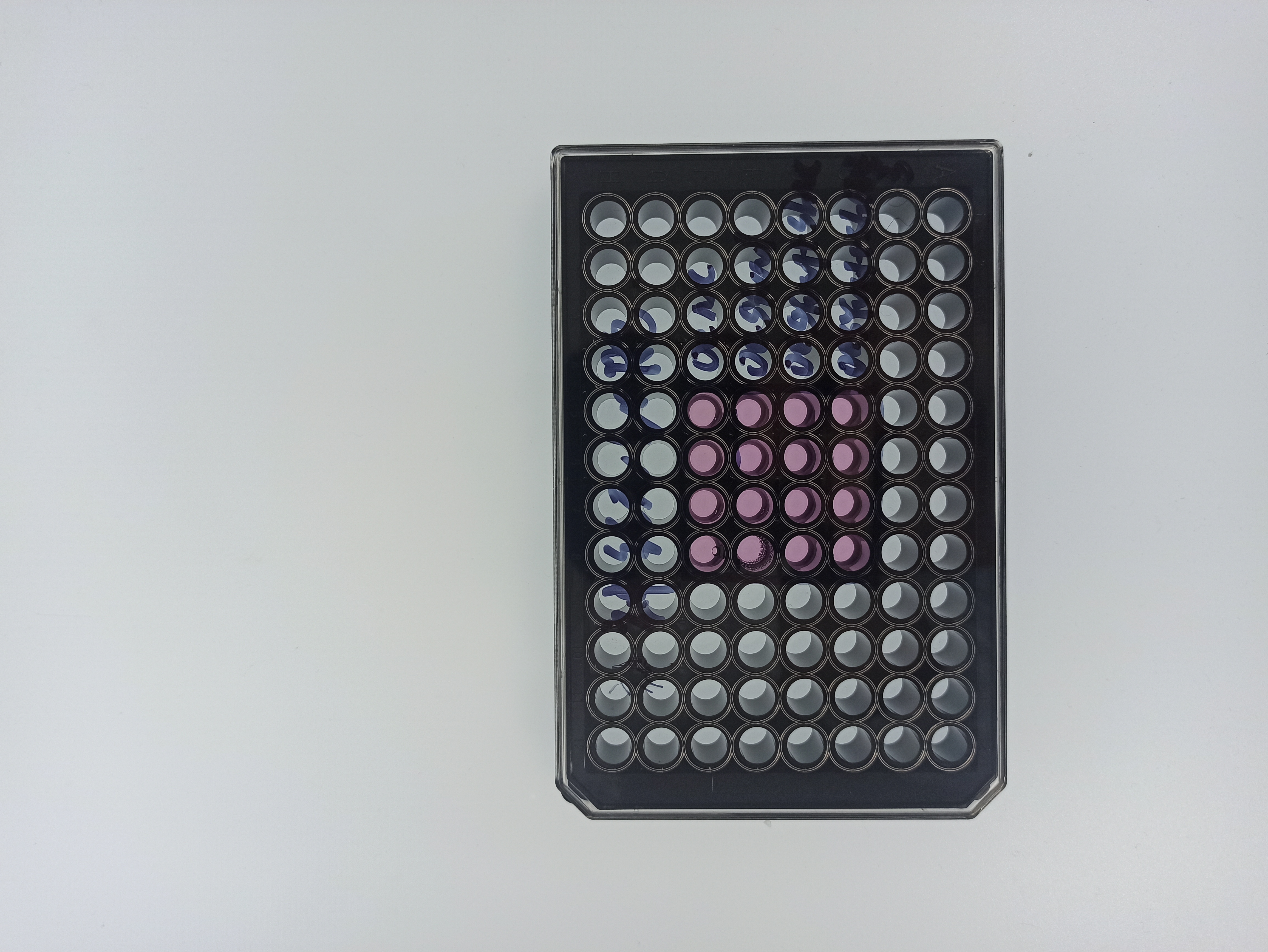

Supplement: Supplementary file 2 — Data S2: Ferroptosis test in Figure S3. [file CPR-57-e13658-s002.zip › Ferroptosis test in Fig S3/Fig S3C Fe2+ Test/Fe2+ blank (1).jpg]

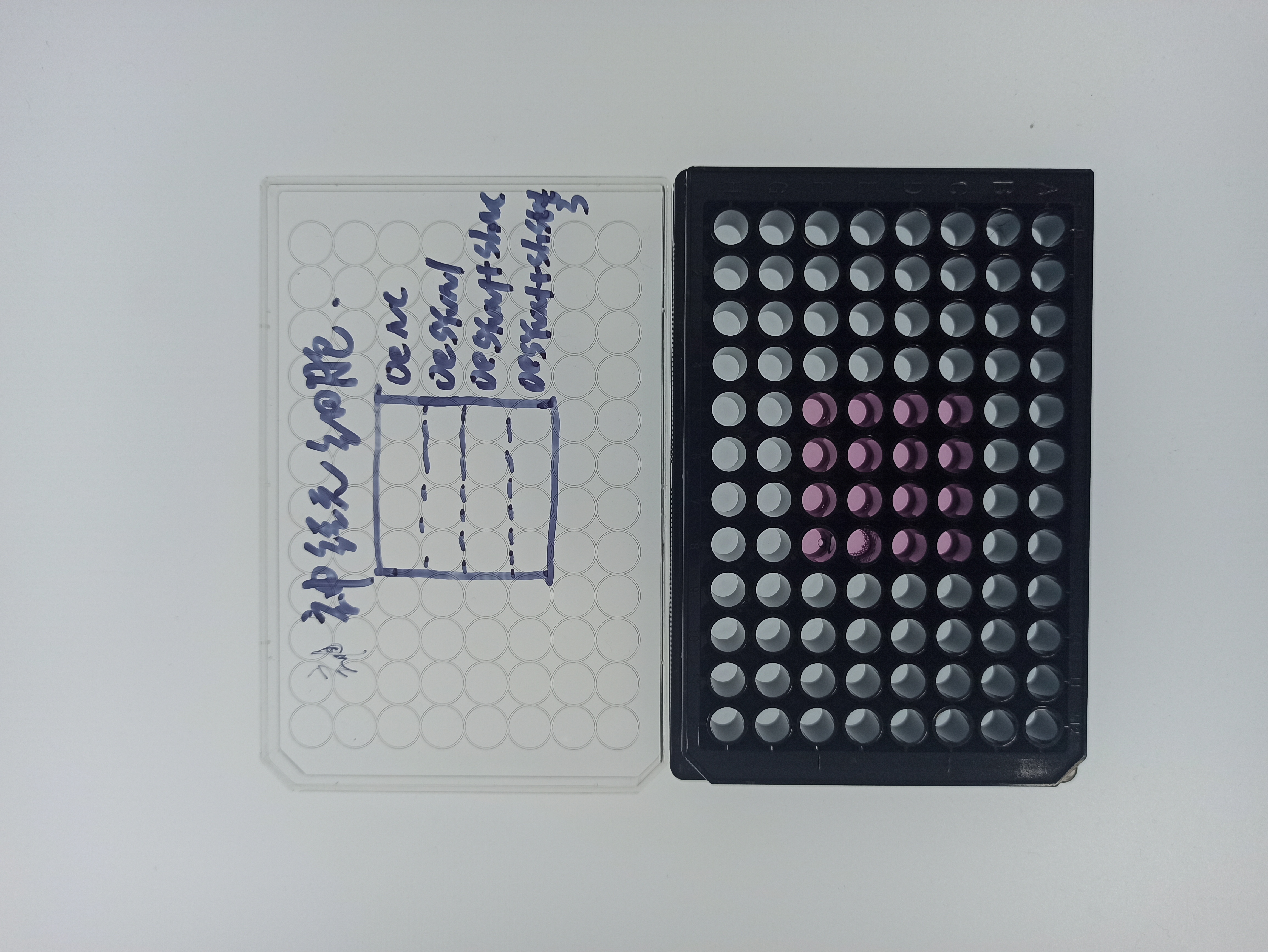

Supplement: Supplementary file 2 — Data S2: Ferroptosis test in Figure S3. [file CPR-57-e13658-s002.zip › Ferroptosis test in Fig S3/Fig S3C Fe2+ Test/Fe2+ blank (2).jpg]
